# First complete chloroplast genomics and comparative phylogenetic analysis of *Commiphora gileadensis* and *C*. *foliacea*: Myrrh producing trees

**DOI:** 10.1371/journal.pone.0208511

**Published:** 2019-01-10

**Authors:** Arif Khan, Sajjad Asaf, Abdul Latif Khan, Ahmed Al-Harrasi, Omar Al-Sudairy, Noor Mazin AbdulKareem, Adil Khan, Tariq Shehzad, Nadiya Alsaady, Ali Al-Lawati, Ahmed Al-Rawahi, Zabta Khan Shinwari

**Affiliations:** 1 Natural and Medical Sciences Research Center, University of Nizwa, Nizwa, Oman; 2 Department of Biotechnology, Quaid-i-Azam University, Islamabad, Pakistan; 3 Plant Genome Mapping Lab, Center for Applied Genetic Technologies, University of Georgia, Georgia, United States of America; 4 Oman Animal & Plant Genetic Resources Center, The Research Council, Muscat, Oman; National Cheng Kung University, TAIWAN

## Abstract

*Commiphora gileadensis* and *C*. *foliacea* (family *Burseraceae*) are pantropical in nature and known for producing fragrant resin (myrrh). Both the tree species are economically and medicinally important however, least genomic understanding is available for this genus. Herein, we report the complete chloroplast genome sequences of *C*. *gileadensis and C*. *foliacea* and comparative analysis with related species (*C*. *wightii* and *Boswellia sacra*). A modified chloroplast DNA extraction method was adopted, followed with next generation sequencing, detailed bioinformatics and PCR analyses. The results revealed that the cp genome sizes of *C*. *gileadensis* and *C*. *foliacea*, are 160,268 and 160,249 bp, respectively, with classic quadripartite structures that comprises of inverted repeat’s pair. Overall, the organization of these cp genomes, GC contents, gene order, and codon usage were comparable to other cp genomes in angiosperm. Approximately, 198 and 175 perfect simple sequence repeats were detected in *C*. *gileadensis and C*. *foliacea* genomes, respectively. Similarly, 30 and 25 palindromic, 15 and 25 forward, and 20 and 25 tandem repeats were determined in both the cp genomes, respectively. Comparison of these complete cp genomes with *C*. *wightii and B*. *sacra* revealed significant sequence resemblance and comparatively highest deviation in intergenic spacers. The phylo-genomic comparison showed that *C*. *gileadensis* and *C*. *foliacea* form a single clade with previously reported *C*. *wightii* and *B*. *sacra* from family *Burseraceae*. Current study reports for the first time the cp genomics of species from *Commiphora*, which could be helpful in understanding genetic diversity and phylogeny of this myrrh producing species.

## Introduction

The family *Burseraceae* comprises 18 genera and about 700 species [1]. The family has pantropical nature and is known for its fragrant resin, such as myrrh and frankincense. The family comprises of timber trees, small trees and shrubs [2,3]. The genus *Commiphora* comprises 190 plant species and distributed in southern Arabia (Yemen, Oman), northeastern Africa (Somalia, Ethiopia, Sudan) and subcontinent (India, Pakistan) [4–6]. The resin obtained from the tree by tapping is widely used in perfume, fragrance and medicinal products [3]. In indigenous medicine, resin based recipes are used for gastrointestinal, arthritis, wounding, obesity, pain and parasitic infections [7]. In the Sultanate of Oman, several *Commiphora* species are reported such as *C*. *gileadensis*, *C*. *foliacea*, and *C*. *habessinica*, [3].

*C*. *gileadensis* is widely known in the Mediterranean basin, especially on border of Oman, Saudi Arabia, Yemen and Somalia [1]. It is also known as balsam and commonly used for production of expensive perfumes [8,9]. Its sap, wood bark and seeds are used for medicinal purposes. Similarly, *C*. *gileadensis* yields in the production of very fragrant gum type resin, when the bark of the tree is damaged [10]. *C*. *gileadensis* was recognized in ancient times as a perfume and incense plant [11]. *C*. *gileadensis* also possess antibacterial properties and the people use it for treatment of infections [12]. *Commiphora* is used for the treatment of an opportunistic fungal infection in many countries of Africa [12]. *C*. *foliacea* was initially considered as endemic to Oman [1], but this specie was also reported in southern coast line of Yemen and Somalia [13,14].

Studying the genomics of ecologically and medicinally important wild trees can help in understanding the tree life, evolution, taxonomy and genetic diversity. In this regard, chloroplast (an important player of photosynthesis) genomics have been widely used in phylogenetic studies due to its maternal inheritance and recombination free nature [15]. The high conserved structure of chloroplast facilitates; primer designing, sequencing and used as a barcode for the identification of plants [16,17]. It contains its own independent genome, which encodes for specific proteins[18]. The genome is circular in structure that varies from 120 kb to 170kb and quadripartite configurations [19]. The chloroplast genome is composed of small single copy (SSC) region and large single copy (LSC) regions, separated by two copies of inverted repeats (IRa and IRb) [19]. They also provide important information in taxonomic and phylogenetic context on basis of differences in the sequences among plant species [20,21]. Chloroplast is haploid, maternally inherited and possess high conservation in gene content, which make it a good choice for studying evolutionary relationship in plants at any taxonomic levels [20]. The first complete chloroplast genome of the angiosperms were reported in tobacco [22]. Advances and rapid evolution in NGS (next-generation sequencing) technologies have made it possible the rapid sequencing of complete chloroplast genome sequences at much cheaper price. Up till now over 2700 cp genome sequences are submitted to National Center for Biotechnology Information (NCBI) including all of major groups of the plant kingdoms. However, still there are numerous economically and medicinally important plants species, which needs to be explored and understood in term of their chloroplast genome structure, organization and genetic evolution. Current study is our first effort to understand the two unexplored species *C*. *gileadensis* and *C*. *foliacea*. We sequenced the cp genomes and performed a detailed comparison with *C*. *wightii* and *B*. *sacra* to understand the genome structure, variation and phylogenetic placements.

## Material and method

### Ethic statement

The leaf samples were collected with care and trees were treated ethically. During sample collection, the local environment was not harmed. Permission was granted by Ministry of Environment, Muscat, Sultanate of Oman to collect leaf samples for research purpose. The current study did not involve endangered or protected species.

### Sample collection

Leaf samples were collected from Wadi Darbaat, Dhofar-Oman (17 31.237’N 55’ 12.923'E). The samples include fresh and young photosynthetic leaves of *C*. *gileadensis* and *C*. *foliacea*. The collected samples were kept immediately in liquid nitrogen and then stored at -80°C until chloroplast DNA extraction.

### Chloroplast DNA extraction and sequencing

Leaf samples of *C*. *gileadensis* and *C*. *foliacea* were cleaned and washed with sterilized water, air dried and kept in dark for 48 hrs in order to reduce the starch content in leaf tissues. Chloroplast DNA was extracted by the protocol of Shi et al, [23] with modifications to remove the traces of resinous content from tissues. The workflow of Ion Torrent S5 Sequencer (Life Technologies, USA) was used for extracted cp DNA sequencing. Chloroplast DNA were enzymatically sheared for 400 bp using the Ion Shear Plus Reagents and library were prepared following the protocol of Ion S5 with Ion Xpress Plus DNA Fragment Library kit. Prepared libraries were checked on Qubit fluorimeter and bioanalyzer (Agilent 2100, CA, USA) for quality check and standardization. Ion One Touch 2 instrument was used for template amplification, post template amplification, whereas the enrichment process was carried out with Ion One Touch ES enrichment system. The sample was loaded onto the Ion S5 Chip and sequencing were performed according to the protocol of Ion Torrent S5.

### Genome assembly

The quality of raw reads were evaluated by using the FastQC [24]. Adapters were removed from both end of the contigs and Platanus_trim (v.1.0.7) [25] with phred score >30 was used to trim high quality reads. The chloroplast genomes of both *Commiphora* species were first *de novo* assembled. In order to get contamination free read of chloroplast genome from mitochondrial and nuclear genomes, the *Commiphora* species genomes paired end reads were obtained by mapping the high quality reads to a selected reference genome of *C*. *wightii* (NC036978) with Bowtie2 (v.2.2.3) [26]. The selected resultant reads were assembled using Spades (v.3.7.1) software [27] and the parameters were set to default. The regions which was uncertain in these genomes such as IR junctions region were picked out from the already published genome of *C*. *wightii* and *B*. *sacra* (NC036978 and NC029420, respectively), to adjust the sequence length, iteration method was used with software MITObim (v.1.8) [28]. The complete genome sequences were deposited in Gene Bank of NCBI, where *C*. *gileadensis* and *C*. *foliacea* were given MH042752 and MH041484 accession numbers, respectively.

### Genome annotation

Chloroplast genomes were annotated by using Dual Organellar Genome Annotator (DOGMA) [29] and BLASTX and BLASTN were used to identify the positions of ribosomal RNAs, transfer RNAs and coding genes, tRNAscan-SE77 software was used to annotate tRNA genes. Furthermore, for manual adjustment, Geneious Pro (v.10.2.3) [30]and tRNAscan-SE [31] were used to compare it with previously reported *C*. *wightii* genome. Similarly, the start and stop codon and intron boundaries were also manually adjusted compared with pre sequenced *C*. *wightii* and *B*. *sacra*. Furthermore, the structural features of both *Commiphora* species cp genome were illustrated using OGDRAW [32]. Similarly, MEGA6 software [33] was used to determine the relative synonymous codon usage and divergence in usage of identical codons. The divergence of these two *Commiphora species* cp genome with other related species were determined by using mVISTA [34] in Shuffle—LAGAN mode and using *C*. *wightii* as a reference genome.

### Repeat identification

REPuter software [35] was used for the identification of palindromic, tandem and forward repeats present in genome. The criterion was minimum >15 base pairs with sequence identity of 90%. SSRs dataset was determined through PHOBOS ver3.3.12 [36] inclusive of attributed sets with (i) mononucleotide repeats ≥10 repeat units (ii) dinucleotide repeats ≥8 repeat units (iii) tri nucleotide and tetra nucleotide repeats ≥4 repeat units, and (iv) penta nucleotide and hexa nucleotide repeats ≥3 repeat units. Tandem Repeats Finder version 4.07 b [37] with default settings was used to determined tandem repeats.

### Sequence-divergence and Phylo-genomic analysis

In this analysis, average-pairwise sequence divergence of complete plastomes and shared genes of *Commiphora* species with related species were determined. Missing and ambiguous gene annotations were confirmed by comparative sequence analysis after a multiple sequence alignment and gene order comparisons using Geneious Pro (v.10.2.3) [30] as reported previously [38,39]. These regions were aligned using MAFFT version 7.222 [40] with default parameters. Pairwise sequence divergence was calculated by selected Kimura’s two-parameter (K2P) model [41]. Similarly, a custom Python script (https://www.biostars.org/p/119214/) and DnaSP 5.10.01 [42], were employed to determine single-nucleotide polymorphisms and *Indel* polymorphisms among the complete genomes respectively. To infer the phylogenetic position of both *C*. *gileadensis* and *C*. *foliacea* within the order Sapindales, 24 cp genomes were downloaded from the NCBI database for analysis. Multiple alignments were performed using complete cp genomes based on conserved structures and gene order [41] and 4 different methods were used to make the trees: Bayesian-inference (MrBayes v3.1.2 [43]), maximum parsimony (PAUP-4.0[44]), maximum-likelihood and neighbour joining (MEGA7.01[33]) according to the methods of Asaf et al [39,45]. For Bayesian posterior probabilities (PP) in the BI analyses, the best substitution model GTR + G model was tested according to the Akaike information criterion (AIC) by jModelTest verion 2102. The Markov Chain Monto Carlo (MCMC) was run for 1,000,000 generations with 4 incrementally heated chains, starting from random trees and sampling 1 out of every 100 generations. The first 30% of trees were discarded as burn-in to estimate the value of posterior probabilities. Furthermore, parameters for the ML analysis were optimized with a BIONJ tree as the starting tree with 1000 bootstrap replicates using the Kimura 2-parameter model with gamma-distributed rate heterogeneity and invariant sites. MP was run using a heuristic search with 1000 random addition sequence replicates with the tree-bisection-reconnection (TBR) branch-swapping tree search criterion. In the second phylogenetic analysis, 72 shared genes from the cp genomes of the twenty-six members of order Sapindales, were aligned using ClustalX with default settings, followed by manual adjustment to preserve reading frames. Similarly, the above4 mentioned phylogenetic inference models were utilized to build trees using 72 concatenated genes, using the same setting as described above and suggested by Asaf et al [45].

## Result and discussion

### Genome features, content and organization

**Fig 1:**
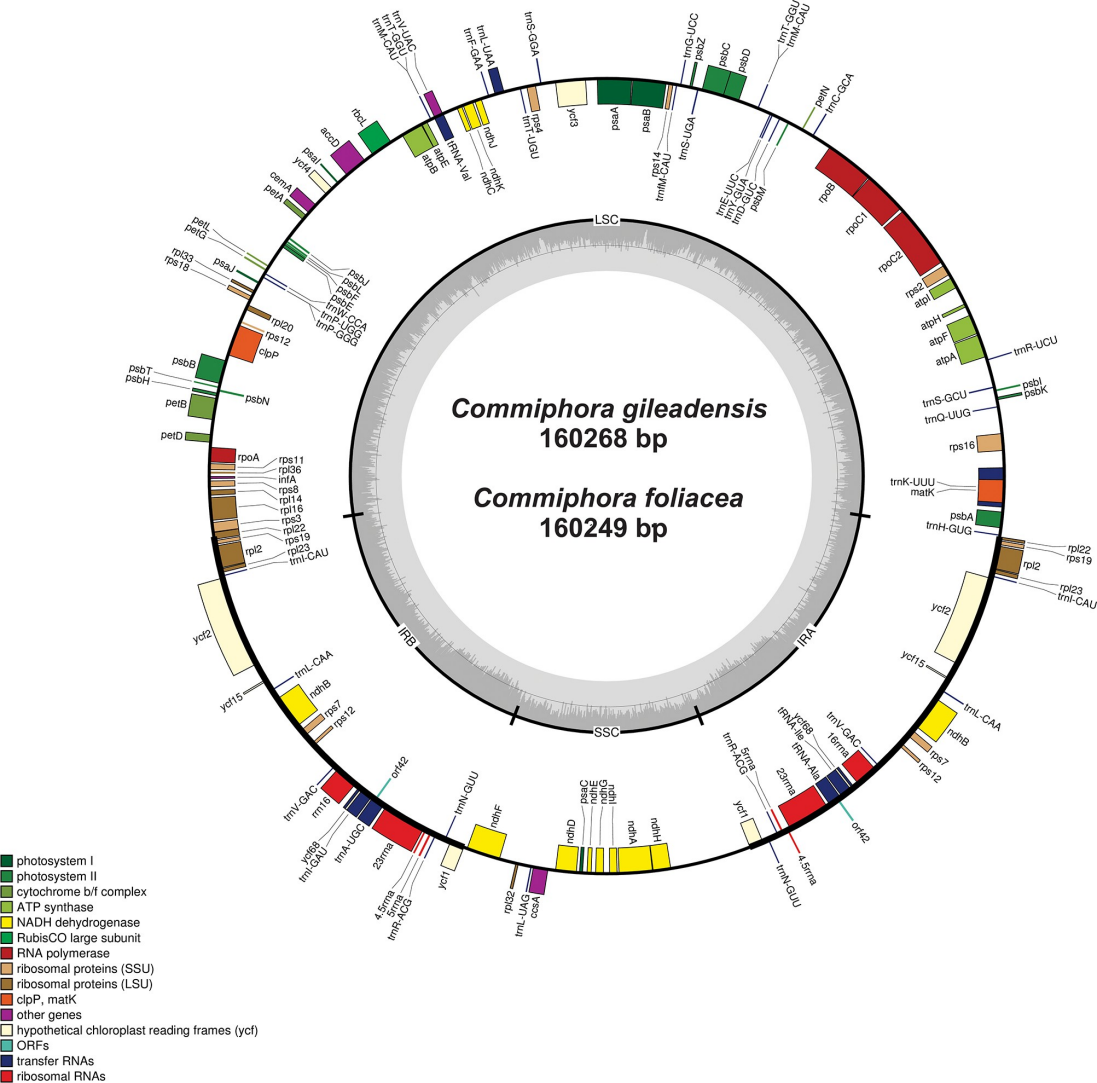
Genome map of the *C*. *gileadensis* and *C*. *foliacea* cp genomes. Thick lines indicate the extent of the inverted repeat regions (IRa and IRb), which separate the genome into small (SSC) and large (LSC) single copy regions. Genes drawn inside the circle are transcribed clockwise, and those outside are transcribed counter clockwise. Genes belonging to different functional groups are color-coded. The dark grey in the inner circle corresponds to the GC content and the light grey corresponds to the AT content.

The chloroplast genomes of *C*. *gileadensis* (MH042752*)* and *C*. *foliacea* (MH041484) were identical to typical angiosperms genomes of 160,268 bp and 160,249 bp, respectively (Fig 1). The size of these cp genomes were almost similar with previously reported chloroplast genome of *B*. *sacra* (160,543 bp) [46], *Azadirachta indica* (160,737 bp) [47], *Citrus sinensis* (160,129 bp) [48] and *Ailanthus altissima* [49], which belong to order Sapindales. Both of these genomes possess the quadripartite structures comprises a pair of inverted repeats (IRa and IRb) separated by small single copy region (SSC) and large single copy region (LSC). The LSC regions in these genomes varies from 87,885 bp to 88,054 bp, SSC varies from 18,746bp to 18,962bp, and the inverted repeat region varies from 26,763bp to 26,807bp (Fig 1). Similarly, the length of LSC, SSC and IR regions was also similar with previously reported genomes for order Sapindales [48,49].

Furthermore, the average GC content of *C*. *gileadensis* and *C*. *foliacea* genomes were found 37.8% which is almost similar to *B*. *sacra* (37.8%) and *C*. *wightii* (38%). The GC content of these cp genomes were also found similar with previously reported *Sesamum indicum* L. which is approximately 38% [50]. The AT content of both the cp genomes were 62.2%. This is in correlation to the other species from order Sapindales, for example *A*. *miaotaiense* (62.12%) [51], *A*. *davidii* (62.10%) [52], *C*. *sinensis* (61.52%)[48] and *P*. *amurense* (61.60%) [53]. Overall, the A+T content of 62.14% in both the cp genomes are closely related to order Sapindales (Table 1).

**Table 1:** Summary of complete *Commiphora gileadensis* and *C*. *foliacea* chloroplast genomes.

Parameters	*C*. *gileadensis*	*C*. *foliacea*	*C*. *wightii*	*B*. *sacra*
**Size (bp)**	160268	160249	156064	160543
**Overall GC contents (%)**	37.9	37.8	38	37.6
**LSC size in bp**	87885	87923	-	88054
**SSC size in bp**	18769	18746	-	18962
**IR size in bp**	26807	26790	-	26763
**Protein coding regions size in bp**	78238	78119	60006	80361
**tRNA size in bp**	2934	2934	2919	2195
**rRNA size in bp**	9050	9050	9394	9050
**Number of genes**	142	140	123	142
**Number of protein coding genes**	95	93	86	93
**Number of rRNA**	8	8	8	8
**Number of tRNA**	39	39	29	39

The GC content was unevenly present in the *C*. *gileadensis* and *C*. *foliacea* cp genomes where it was low (32.3 and 32.4%, respectively) in the SSC regions, high (42.9%) in IR regions and moderate (35.8%) in the LSC regions. In synergy to the previously published reports on cp genomes, the presence of ribosomal RNA (rRNA) sequences enhance the GC contents in the IR regions [54–56]. In addition, about 43.72% of *C*. *gileadensis* and 46.91% of *C*. *foliacea* cp genomes were found noncoding. In case of coding regions, the protein coding genes were 48.81 and 45.62%, tRNA genes were 1.83 and 1.83%, and rRNA genes were 5.64 and 5.64% found in the *C*. *gileadensis* and *C*. *foliacea* cp genomes, respectively.

The total coding DNA sequences (CDSs) of *C*. *gileadensis* and *C*. *foliacea* were 78,238 bp and 73,119bp in size which encodes 94 and 93 genes respectively (S1 Table). This also includes 26,078 and 24,273bp codons respectively (S2 Table). Similarly, the codon-usage frequency of the both *C*. *gileadensis* and *C*. *foliacea* cp genomes were determined on the basis of protein—coding and tRNA- related gene sequence (S3 Table, S4 Table). Like previously reported cp genomes, the cysteine (1.2%) and leucine (10.3%) were the least and most commonly encoded amino acids [39,54]. Furthermore, The AT contents of both C. *gileadensis* and *C*. *foliacea* cp genomes at the 1^st^, 2^nd^, and ^3rd^ codon position of CDS were 54.6 and 55.1%, 61.4 and 58.4%, and 65.99 and 67.3%, respectively (S2 Tablehttp://journals.plos.org/plosone/article?id = 10.1371/journal.pone.0182281 - pone-0182281-t003). This is in correlation with previous reports showing that the terrestrial plant’s cp genome with highest AT-content at the 3^rd^ codon-position [54,57]

The total number of genes in the *C*. *gileadensis* and *C*. *foliacea* were 140 and 141 respectively, in which 94 and 93 genes were protein coding genes, while 39 were tRNAs and 8 were rRNAs genes. Similar results were reported in previous reported cp genomes of *B*. *sacra* has 142 genes [46], *A*. *miaotaiense* has 137 [51], *A*. *wangii* has 135 [58], *A*. *buergerianum* has 134 [59], and in *Meliaceae species* has 112 genes [60], which is from the same order Sapindales [51]. *Camellia* species contains 146 genes [60]. The protein-coding genes present in *C*. *gileadensis and C*. *foliacea* cp genomes include twelve genes-encoding small-ribosomal proteins (*rps2*, *rps3*, *rps4*, *rps7*, *rps8*, *rps11*, *rps12*, *rps14*, *rps16*, *rps18*, *rps19*), 9 genes-encoding large ribosomal proteins (*rpl2*, *rpl14*, *rpl16*, *rpl20*, *rpl22*, *rpl23*, *rpl32*, *rpl33*, *rpl36*), 10 genes of photosystem-II, five genes-encoding photosystem-I components, and 6 genes (*atpA*, *atpB*, *atpE*, *atpF*, *atpH*, *atpI*) ATP-synthase and electron-transport chain components (S1 Table). Similarly, the chloroplast genomes of *C*. *gileadensis* and *C*. *foliacea* contains introns containing genes. There were 11 genes containing intron inclusive of nine which have single-intron and 3 (*clpP*, *ycf3* and *rps12*) which have two introns (Table 2). These results are similar with previously reported cp genome of angiosperms. The smallest intron in both *C*. *gileadensis* and *C*. *foliacea* cp genoemes were 518bp and 526 bp respectively, whereas the longest intron was determined in *trnK-UUU* (2507 bp) in both cp genomes that included the entire *matK* gene. Introns can be a useful tool for successful transformational effectiveness and play a vital role in the regulation of gene expression [61]. Like other angiosperms cp genomes, *rps12* gene was unequally distributed, with single copy of its 3′ exon/intron, located at the IR regions and 5′-exon, located in the LSC region. A similar correlation in the results were observed in previously reported cp genomes of *C*. *platymamma* [62], *C*. *aurantiifolia* [63] and *Dipteronia* species [62]. Moreover, there are 4 ribosomal RNA genes and 30 transfer RNA genes. The *infA gene*, which code for transcription factor of initiation was present in both *Commiphora species*, while it is absent in *Citrus sinensis* (L.) cp genome [64].

**Table 2:** The genes with introns in the *C*. *gileadensis (C*. *g)* and *C*. *foliacea (C*. *f)* chloroplast genome and the length of exons and introns.

Gene	Location	Exon I (bp)		Intron 1 (bp)		Exon II (bp)		Intron II (bp)		Exon III (bp)	
		*C*. *g*	*C*. *f*	*C*. *g*	*C*. *f*	*C*. *g*	*C*. *f*	*C*. *g*	*C*. *f*	*C*. *g*	*C*. *f*
*atpF*	LSC	159	159	719	718	438	438				
*petB*	LSC	6	6	753	749	648	645				
rpl2*		393	393	662	662	432	435				
*rpl16*	LSC	9	9	1088	1086	411	411				
*rps16*	LSC	48	48	871	864	225	225				
*rpoC1*	LSC	432	435	791	791	1620	1620				
rps12*		114	114	-	-	243	243				
*clpP*	LSC	69	69	873	862	267	267	663	656	218	228
*ndhA*		553	553	1109	1104	539	534				
ndhB*		777	777	681	681	753	756				
*ycf3*	LSC	129	129	719	716	228	228	768	768	150	150
trnA-UGC*	IR	38	38	805	803	35	35				
trnI–GAU*	IR	42	42	956	957	35	35				
*trnL-UAA*	LSC	37	37	518	526	50	50				
*trnK -UUU*	LSC	37	37	2507	2507	35	35				

C. g = C. gileadensis, C. f = C. foliacea

### Expansion and contraction of IRs

**Fig 2:**
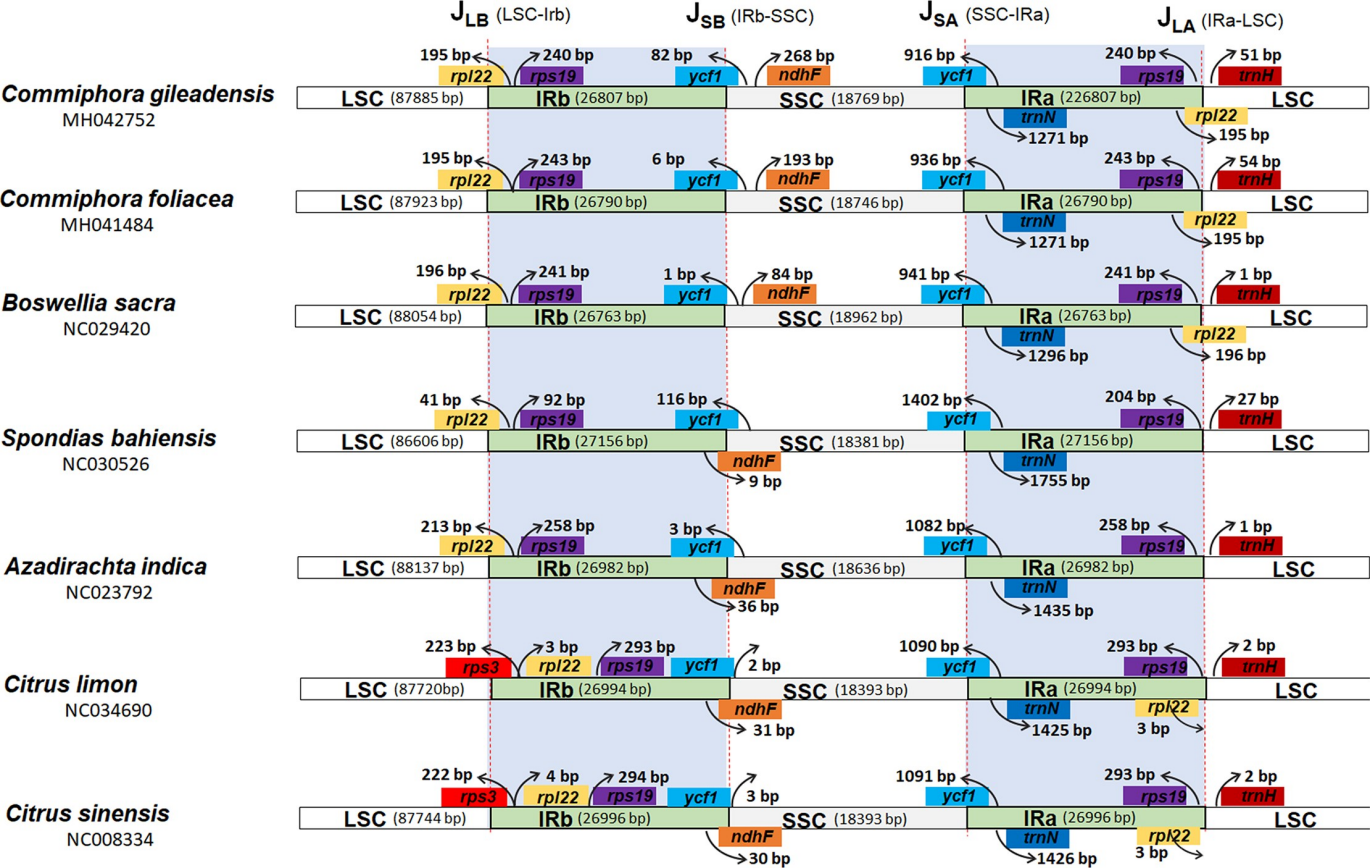
Distance between adjacent genes and junctions of the small single-copy (SSC), large single-copy (LSC), and two inverted repeat (IR) regions among seven plastid genomes within order Sapindales. Boxes above and below the main line indicate the adjacent border genes. The figure is not to scale regarding sequence length, and only shows relative changes at or near the IR/SC borders.

Expansion and contraction of the IR (a&b) repeats were compared among different species belonging to order Sapindales. The chloroplast genomes of angiosperm are highly conserved, but there is still some variation due to contraction or expansion of SSC and IR boundary region [64]. Due to these contraction and expansion, the size variation and rearrangement occurs in the LSC/SSC/IRA/IRB [64]. In this study we carried out a detail comparison of 4-junctions (JLA, JLB, JSA, and JSB) between LSC and SSC regions and both the IRa and IRb regions of the *C*. *gileadensis* and *C*. *foliacea* species and five other species from order Sapindales were performed (Fig 2). Despite the similar IR regions lengths of *C*. *gileadensis* and *C*. *foliacea* with other related species, some contraction and expansion were determined with the IR regions ranging from 26,763 bp in *B*. *sacra* to 27,156 bp in *Spondias bahiensis*. The genes present at starts and end of IR-regions were partly repeated, including 195 bp of *rpl22* in both *C*. *gileadensis* and *C*. *foliacea*, 196 bp in *B*. *sacra*, 4bp and 213 bp in *S*. *bahiensis* and *A*. *indica* respectively. However, in *Citrus lemon* and *Citrus sinensis* the duplicated gene was *rps3* which is located 223 and 222 bp in inverted repeat region from JLB (Fig 2). Correspondingly, the *ycf1* gene which is considered as a hypothetical is duplicated partially, 916 bp and 936 bp in *C*. *gileadensis* and *C*. *foliacea*, 941bp, 1402 bp, 1082bp, 1090 bp and 1091 bp in *B*. *sacra*, *S*. *bahiensis*, *A*. *indica*, *C*. *lemon and C*. *sinesis* respectively. J _LA_ is positioned between *trnH* and *rps19*, whereas the deviation in gaps between J_LA_ and *rps19* range from 240 to 293 bp throughout compared species. Similarly, the detachment in *C*. *gileadensis* and *C*. *foliacea* was 240 bp and 243 bp correspondingly. The distance between *trnH* and J_LA_ was 51 bp and 54 bp in *C*. *gileadensis* and *C*. *foliacea*, which is 1 bp in *B*. *sacra* and *A*. *indica*. Furthermore, variation was observed in the location of *ndhF* genes which is present at 268 bp, 193 bp and 84 bp away from J_SB_ in SSC regions in *C*. *gileadensis*, *C*. *foliacea* and *B*. *sacra* cp genomes. However, in other four species cp genomes *ndhF* was located at the junction of IRb-SCC. Furthermore, there is 76 bp variation was observed in location of *ycf1* gene at J_SB_ border in both *C*. *gileadensis* and *C*. *foliacea*. However, in *B*. *sacra* cp genome this distance was calculated 1 bp away from J_SB_ border [46]. Similar to previously reported cp genome from Sapindales these cp genomes having well-maintained genomic structure in term of cp genome length, IR regions, gene order and gene numbers [49]. However, some of the deviation in sequence might be due to the result of boundary contraction and expansion between the boundaries of IR and single copy regions among different plant species as reported by Wang et al. [64].

### Structural variation in genomic regions

**Fig 3:**
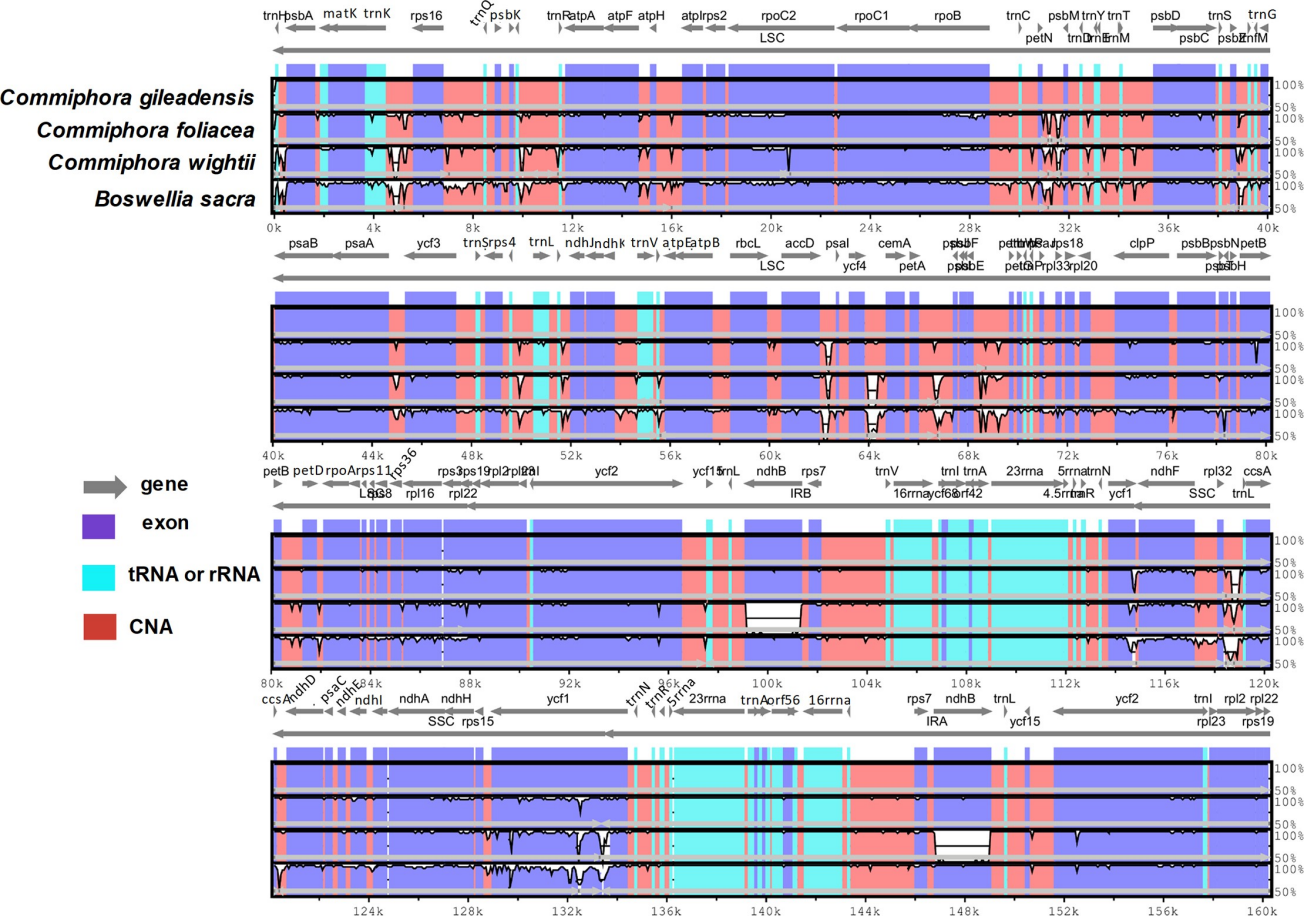
Visual alignment of plastid genomes from *C*. *gileadensis* and *C*. *foliacea* with previously reported *C*. *wightii and B*. *sacra*. VISTA-based identity plot showing sequence identity among seven species, using *C*. *gileadensis* as a reference.

**Fig 4:**
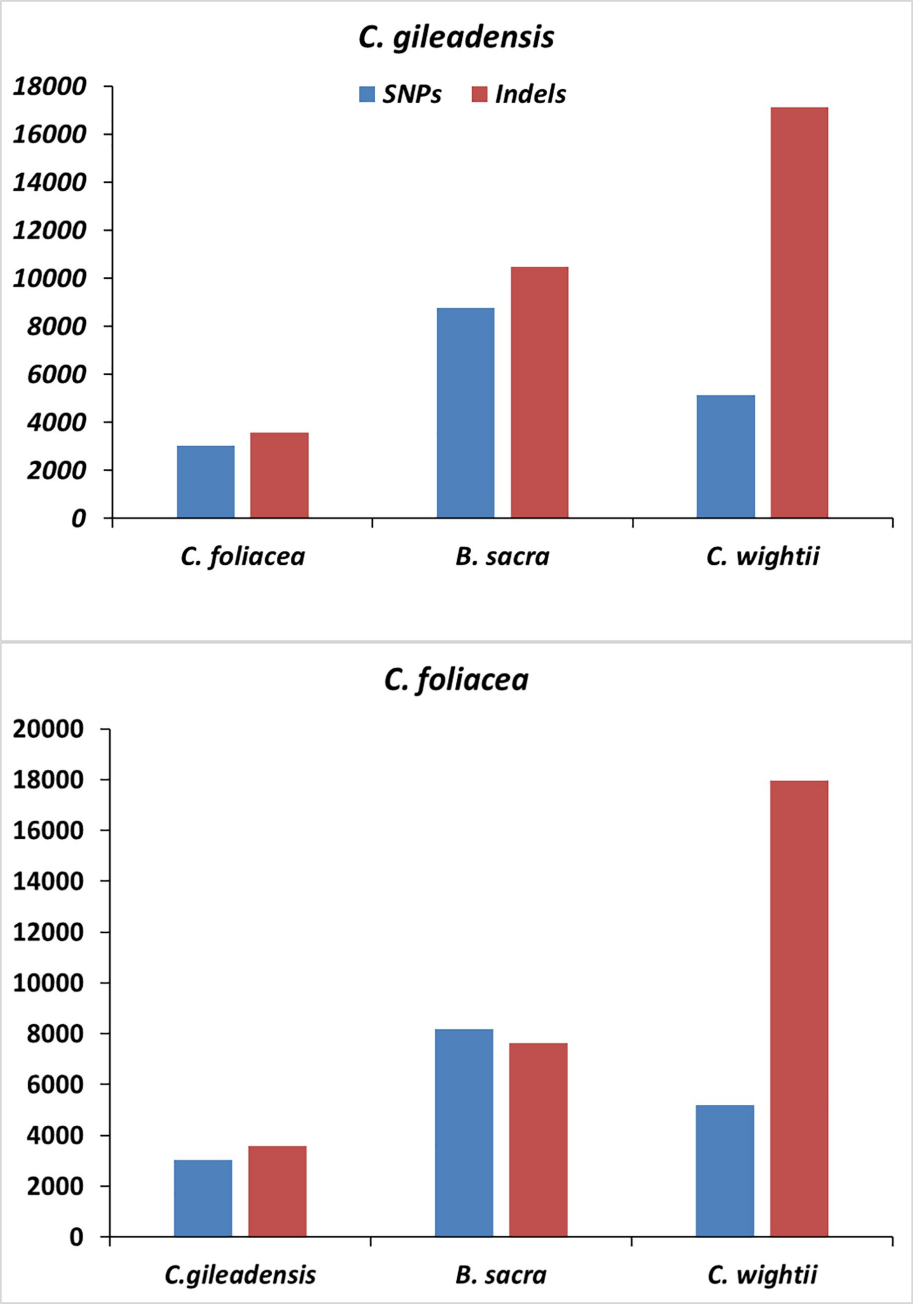
Single nucleotide polymorphism (SNP) and insertion and deletion in *Commiphora* species with related species *Commiphora wightii* and *Boswellia Sacra*.

In order to determine the sequence divergence among the four chloroplast genomes viz. *C*. *gileadensis*, *C*. *foliacea*, *C*. *wightii* and *B*. *sacra*, the annotation of *C*. *gileadensis* cp genome was used as a reference for determination of the sequence similarity in the cp genomes of the three species through mVISTA program (Fig 3). The results showed that high degree of synteny and comparatively lower sequence similarity were noted among these cp genome of these four species especially in *rpoC2*, *rpoB*, *petB*, *psaB*, *ndhB*, *ndhF*, *ccsA*, *ycf1*, *ycf2*, *rpl22* and *atpF* genes (Fig 3). Furthermore, like previous reported genomes the LSC and SSC regions were more divergent as compared to IR regions in the compared species and less similarity in the coding region were observed. Similarly, various deviating regions included *matK*, *ycf3-psaA*, *clpP*, *accD*, *atpF*, *rpoC1*, *petA-psbJ*, *ycf1-rps15*, *rps19* and *ndhF* were reported previously in various cp genomes [54,56]. Differences in the coding regions were similar in this study to the previously analyzed cp genome by Kumar et al. [33]. Similarly, for the shared genes the average pairwise sequence differentiation was calculated among these four species (Fig 3 and S9 Table). The results revealed that the 13 most divergent genes among these genomes were *infA*, *rps8*, *rpl32*, *rpl22*, *rpl16*, *psaI*, *ndhH*, *ndhG*, *matK*, *ccsA*, *atpH*, *accD and psbN*. The *rpl22* gene showed the greatest average sequence divergence (0.029), after that *rps3* (0.028), *ndhH* (0.027), and *ccsA* (0.020), majority of these were located in the LSC region. Similar results were observed in previously reported angiosperm cp genomes [56,65]. Furthermore, comparison of the cp genome of *C*. *gileadensis* with *C*. *foliacea*, *C*. *wightii* and *B*. *sacra* revealed 3,032, 8,787 and 5,120 SNPs as well as 3,580, 10,460 and 17,122 *Indels* respectively (Fig 4). Similarly, the *C*. *foliacea* cp genome also showed 8,194 and 5,182 SNPs while 7,632 and 17,970 *Indel* with *C*. *wightii* and *B*. *sacra* respectively. These Results shows that even the most conserved genome possesses some interspecific mutations which provides an important information in analyzing the phylogenetic and genetic diversity among the species [56].

### SSR Polymorphism in the cp Plastomes

**Fig 5:**
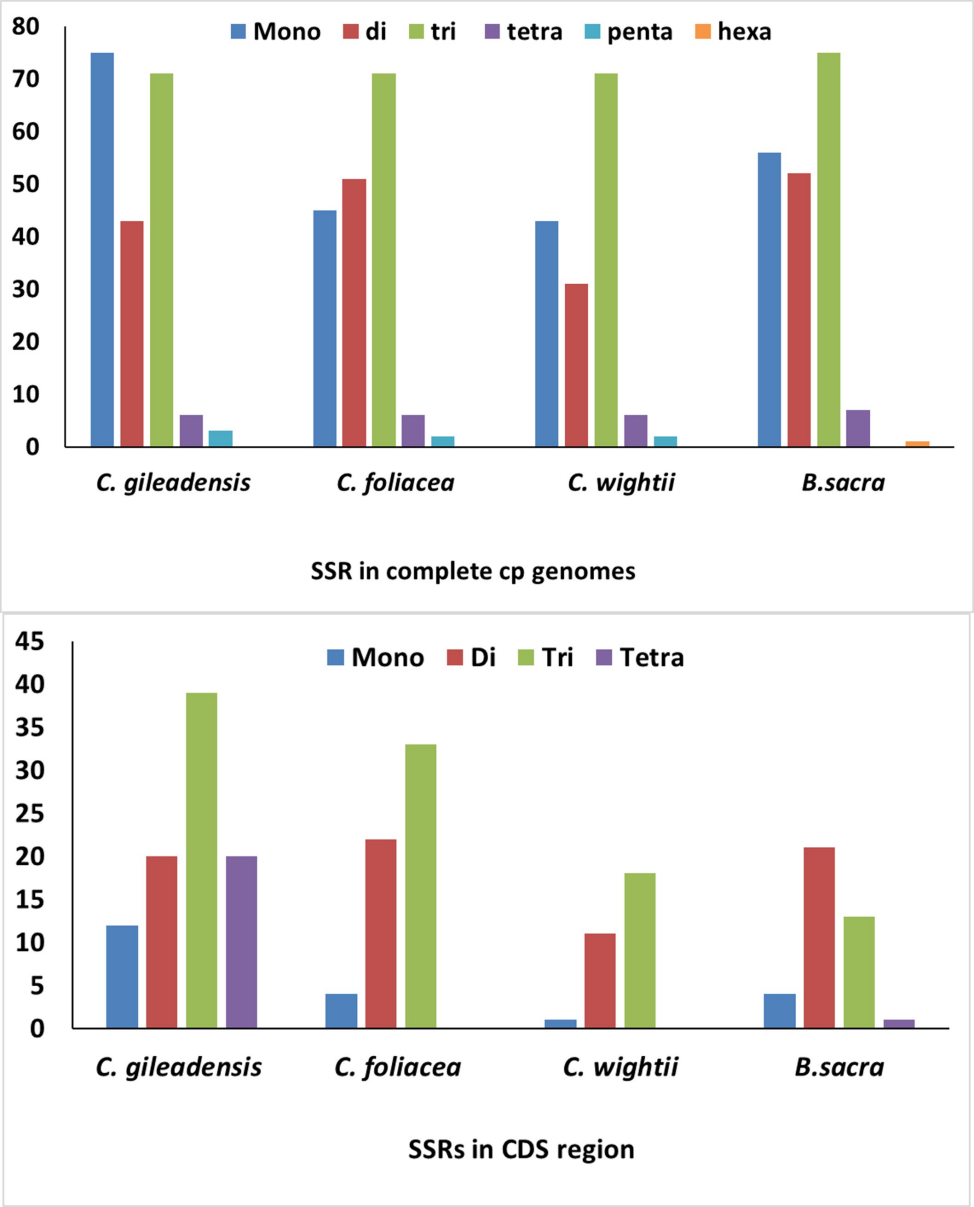
Analysis of simple sequence repeat (SSR) in the *C*. *gileadensis* and *C*. *foliacea* plastid genome. **A**, Number of SSR types in complete genome, coding, and non-coding regions; **B**, Frequency of identified SSR motifs in different repeat class types.

Diversity exist in the copies of SSRs present in the chloroplast genome and these SSRs are vital molecular markers in the plant evolutionary, population genetics and studying the ecology of the plants [66]. In the present study, we detected complete SSRs in *C*. *gileadensis*, *C*. *foliacea* cp genomes together with *C*. *wightii* and *B*. *sacra* (Fig 5) and detail SSR analysis of C. gileadensis, *C*. *foliacea*, *C*. *wightii* and *B*. *sacra* were also performed (S5 Table, S6 Table, S7 Table, S8 Table). Specific parameters were set for the SSRs present in genome because SSR of more than 10bp are liable to slip strand mispairing, which is considered to be the basic reason for SSR polymorphism. [67–69]. The results reveled a total of 196, 175, 153 and 191 SSRs in the *C*. *gileadensis*, *C*. *foliacea*, *C*. *wightii* and *B*. *sacra* cp genomes, respectively. The majority of SSRs 75 (38.2%) in *C*. *gileadensis* cp genome was mono-nucleotide repeat motifs. However, in other three cp genome the majority of SSRs were tri nucleotides motif, varying from quantity from 71 (40.57%) in *C*. *foliacea* to 75 (39.26%) in *B*. *sacra*. Tri-nucleotide repeat motif was found the second most common 69 (35.2%) in *C*. *gileadensis*. Using our search criterion, 3, 2 and 2 penta nucleotide were detected in *C*. *gileadensis*, *C*. *foliacea and C*. *wightii* cp genome respectively. However, in hexa nucleotide was only detected in *B*. *sacra* cp genome. Furthermore, in *C*. *gileadensis* and *C*. *foliacea*, most common mononucleotide SSRs are A (93.33% and 94.1%) motif, respectively. Approximately, 52% and 67.3% of SSRs are sited in non-coding regions, 2.04% and 5.71% are located in rRNA sequences in both *C*. *gileadensis* and *C*. *foliacea* respectively. These results suggest that SSRs are irregularly disseminated in the chloroplast genome and provides valuable information to select the effective molecular markers for spotting inter and intra specific polymorphisms [70–72]. The abundance of ‘A’ and ‘T’ nucleotide in the cp genomes as compared to ‘G’ and ‘C’ is due to the fact that mono and dinucleotide is only consist of ‘A’ and ‘T’ nucleotide which contributes to the bias in the cp genome base composition [68]. The finding from these *Commiphora* genomes reveals that SSRs in the cp genomes are normally composed of polyadenine (polyA) or polythymine (polyT) repeats and irregularly contains the tandems guanine (G) or cytosine (C) repeats [73], which is similar to the previous results thus a possible reason for AT richness [46,55,56]. The presence of SSRs in cp genomes will give useful information for primer designing used for phylogeography and population structure at specie level or SSRs can also be used for obtaining useful and important information used for phylogenetic relationship and population genetics [74]. Previously reported *D*. *viscoa* contains 249 SSRs, having the mononucleotide SSRs in highest number followed by tri nucleotide repeats [74,75]. The cp genome of *globe Artichoke* contains 127 repeats is lesser than our findings [76].

### Repeats analysis of *Commiphora* plastomes

**Fig 6:**
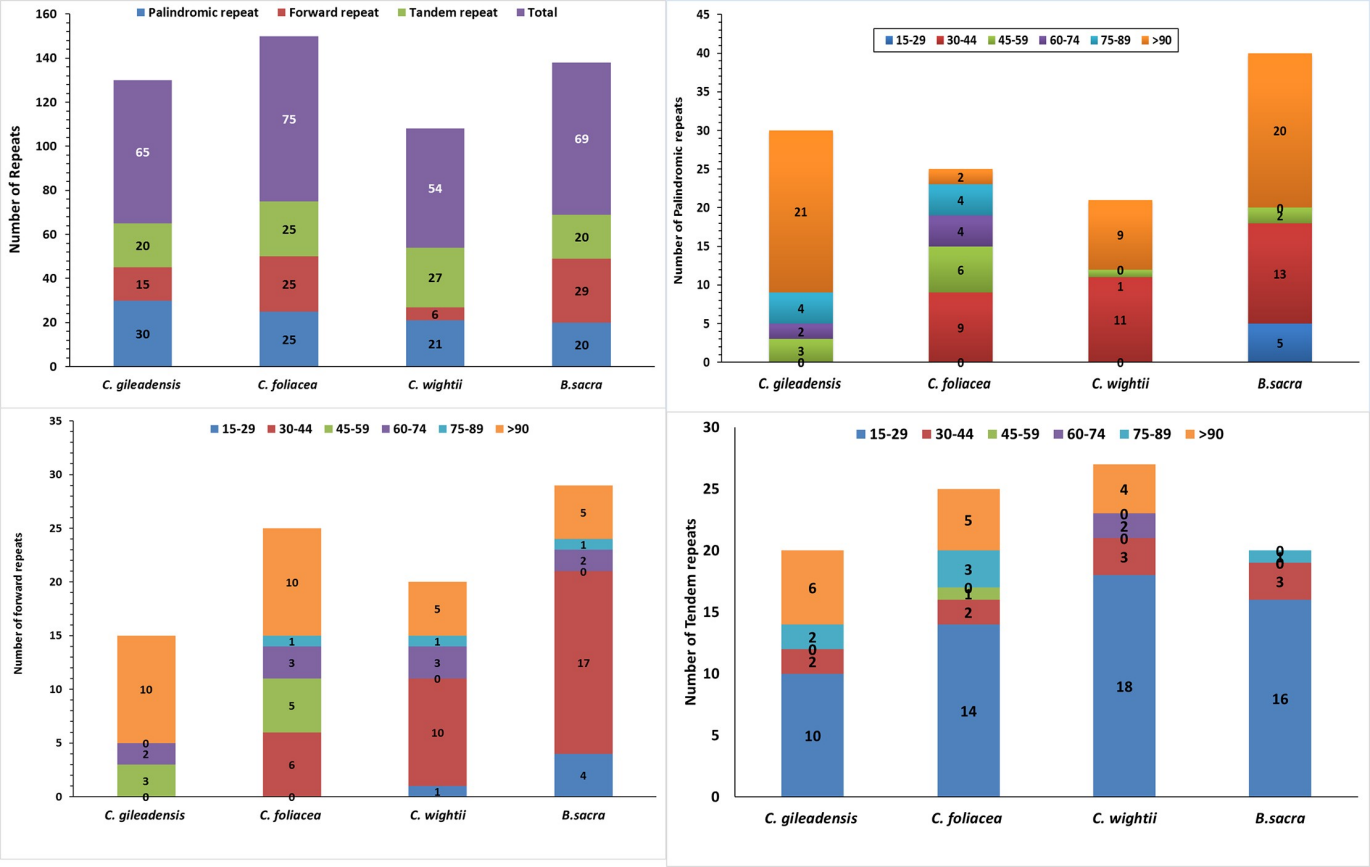
Analysis of repeated sequences in *C*. *gileadensis* and *C*. *foliacea* plastid genome. Totals of three repeat types, Frequency of palindromic repeats by length, Frequency of forward repeats by length and Frequency of tandem repeats by length.

Repetitive sequences in the plastomes plays role in the rearrangement of genomes which provide an important information about phylogenetic studies [50,77] From the previously analyzed cp genomes it is evident that for the induction of indels and substitutions these repeat sequence is essential. Additionally, analysis of different cp-genomes exposed that repeat sequence is important to produce indels/substitutions [78]. Similarly, in our study repeat analysis of the *C*. *gileadensis* and *C*. *foliacea* identified 30 and 25 palindromic repeat, 15 and 25 forward, 20 and 25 tandem repeat respectively. Similarly, 21 and 20 palindromic repeats, 27 and 20 tandem repeats were spotted in *C*. *wightii* and *B*. *sacra* respectively. However, in *C*. *wightii* only 6 forward repeats were detected while in *B*. *sacra* it was 29 in number. Overall 65 and 75 repeats of different length were found in both *C*. *gileadensis* and *C*. *foliacea*, respectively. In *C*. *gileadensis* four palindromic repeats were 75-89bp and 21 repeats were > 90 length. However, in *C*. *foliacea* the number of >90 repeats were less and only 2 palindromic repeats were found. On the other hand, among the forward repeats 10 repeats of >90 bp were detected in both *C*. *gileadensis* and *C*. *foliacea* cp genome (Fig 6). Earlier reports recommend that deviation in sequences and genome arrangement occur due to the slipped-strand mispairing and inappropriate recombination of repetitive sequences [77,79]. Moreover, the occurrence of the repeats shows that this locus is a key hots-pot for re-configuration of the genome [50,80]. Also, the Information from these repeats are a source of valuable information for constructing genetic markers for population studies and phylogenetic analysis [50].

### Phylogenomic analysis

**Fig 7:**
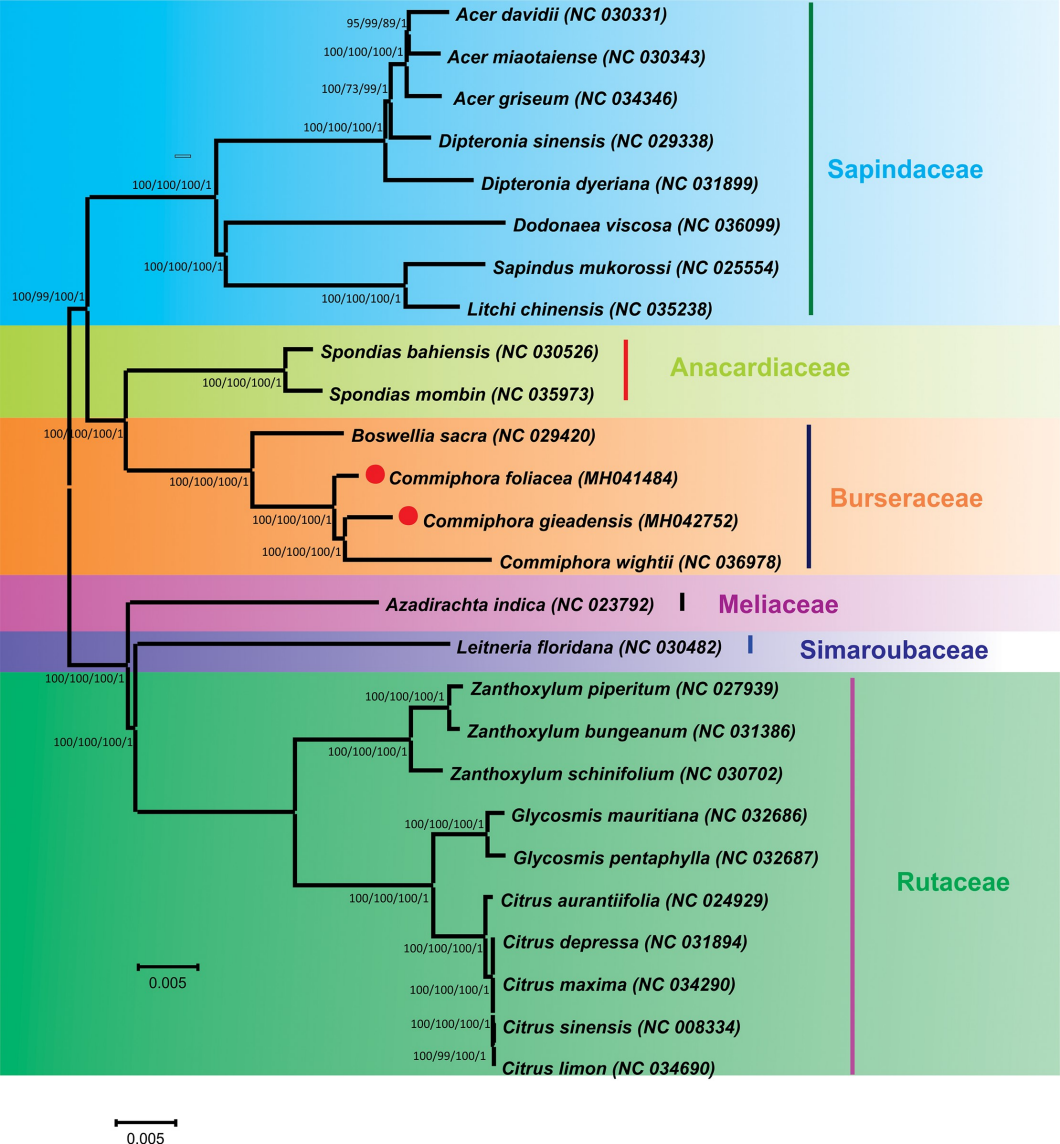
Phylogenetic trees of *C*. *gileadensis* and *C*. *foliacea* within order Sapindales. The entire genome dataset was analyzed using four different methods: Bayesian inference (BI), maximum parsimony (MP), maximum likelihood (ML), and neighbor-joining (NJ). Numbers above the branches represent bootstrap values in the MP, ML, and NJ trees and posterior probabilities in the BI trees, whereas the number below the branches represents branch length. The red dot represents the position of *C*. *gileadensis* and *C*. *foliacea*.

Several aspects of *Commiphora* natural history have impeded efforts to resolve its species-level taxonomy and investigate its systematic biology [81]. Previously, the two species have examined species-level phylogenetic relationships in *Commiphora* and tested the monophyly of some of these infrageneric taxonomic groups [5,82]. Gostel et al. [83] reconstruct phylogenetic relationship in *Commiphora* species using genes from nuclear as well as from chloroplast genome. However, hypothesis regarding higher level relationship among *Commiphora* specie are similarly unresolved [83]. To resolve the phylogenetic relationship among different species, the complete chloroplast genome sequencing provides more detailed information about the phylogenetics [84,85]. Therefore, in this study the phylogenetic position of both *C*. *gileadensis* and *C*. *foliacea* within order Sapindales was established by analyzing the complete cp genomes (Fig 7 and S1) and 72 shared genes (form all twenty-six species). Phylogenetic analysis using MP, BI, NJ and ML methods were performed. The results revealed that both complete cp genomes and 72 shared genes of *C*. *gileadensis* and *C*. *foliacea* contain the same phylogenetic signals and generated phylogenetic trees with identical topologies (Fig 6, S1 Fig). The results show that both *C*. *gileadensis* and *C*. *foliacea* form a single clade with previously reported *C*. *wightii* and *B*. *sacra* from family *Burseraceae* with high BI and bootstrap support values (Fig 7, S1 Fig). The tree topology showed that these four species from family *Burseraceae* are more closely related to *Spondias* species from Family *Anacardiaceae* and *Azadirachta indica* from *Meliaceae* (Fig 7, S1 Fig). Furthermore, the phylogenetic analysis validated the relationship inferred from the phylogenetic work reported by Saina et al. [86] that the families *Burseraceae* and *Anacardiaceae* formed a sister group/clade, which further branched forming sister clade with *Meliaceae*, *Rutaceae*, *Simaroubaceae* and *Sapindaceae* families. Therefore, for future phylogenetic studies must incorporate additional species for better understanding of *Commiphora* species evolution and phylogeny. This study offers a basis for future phylogenetic of family *Burseraceae*.

## Supporting information

S1 Fig Phylogenetic trees of *C. gileadensis* and *C. foliacea* within order Sapindales. The 72 shared gene dataset was analyzed using four different methods: Bayesian inference (BI), maximum parsimony (MP), maximum likelihood (ML), and neighbor-joining (NJ). Numbers above the branches represent bootstrap values in the MP, ML, and NJ trees and posterior probabilities in the BI trees, whereas the number below the branches represents branch length. The red dot represents the position of *C*. *gileadensis* and *C*. *foliacea*.
(TIF)

S1 Table Genes in the sequenced *C. gileadensis* and *C. foliacea* chloroplast genome. (DOCX)

S2 Table Base compositions in *C. gileadensis* (*C. g*), *C. foliacea* (*C. f*), *C. wightii* (*C. w*) and *B. sacra* (*B. s*) cp genomes. (DOCX)

S3 Table The codon–anticodon recognition pattern and codon usage for the *C. gileadensis* chloroplast genome. (DOCX)

S4 Table The codon–anticodon recognition pattern and codon usage for the *C. foliacea* chloroplast genome. (DOCX)

S5 Table Simple sequence repeats (SSRs) in the *C. wightii* chloroplast genome. (DOCX)

S6 Table Simple sequence repeats (SSRs) in *C. gileadensis* chloroplast genome. (DOCX)

S7 Table Simple sequence repeats (SSRs) in *C. foliacea* chloroplast genome. (DOCX)

S8 Table Simple sequence repeats (SSRs) in *Boswellia sacra* chloroplast genome. (DOCX)

S9 Table Pairwise distance of 77 shared genes in three *Commiphora* species (*Commiphora gileadensis*, *Commiphora foliacea*, *Commiphora wightii*) with *Boswellia sacra*. (XLS)
