# Supplementary figures and images for: First complete chloroplast genomics and comparative phylogenetic analysis of Commiphora gileadensis and C. foliacea: Myrrh producing trees
Source: PLoS One. 2019 Jan 10;14(1):e0208511. doi: 10.1371/journal.pone.0208511 (PMC6328178; doi:10.1371/journal.pone.0208511)

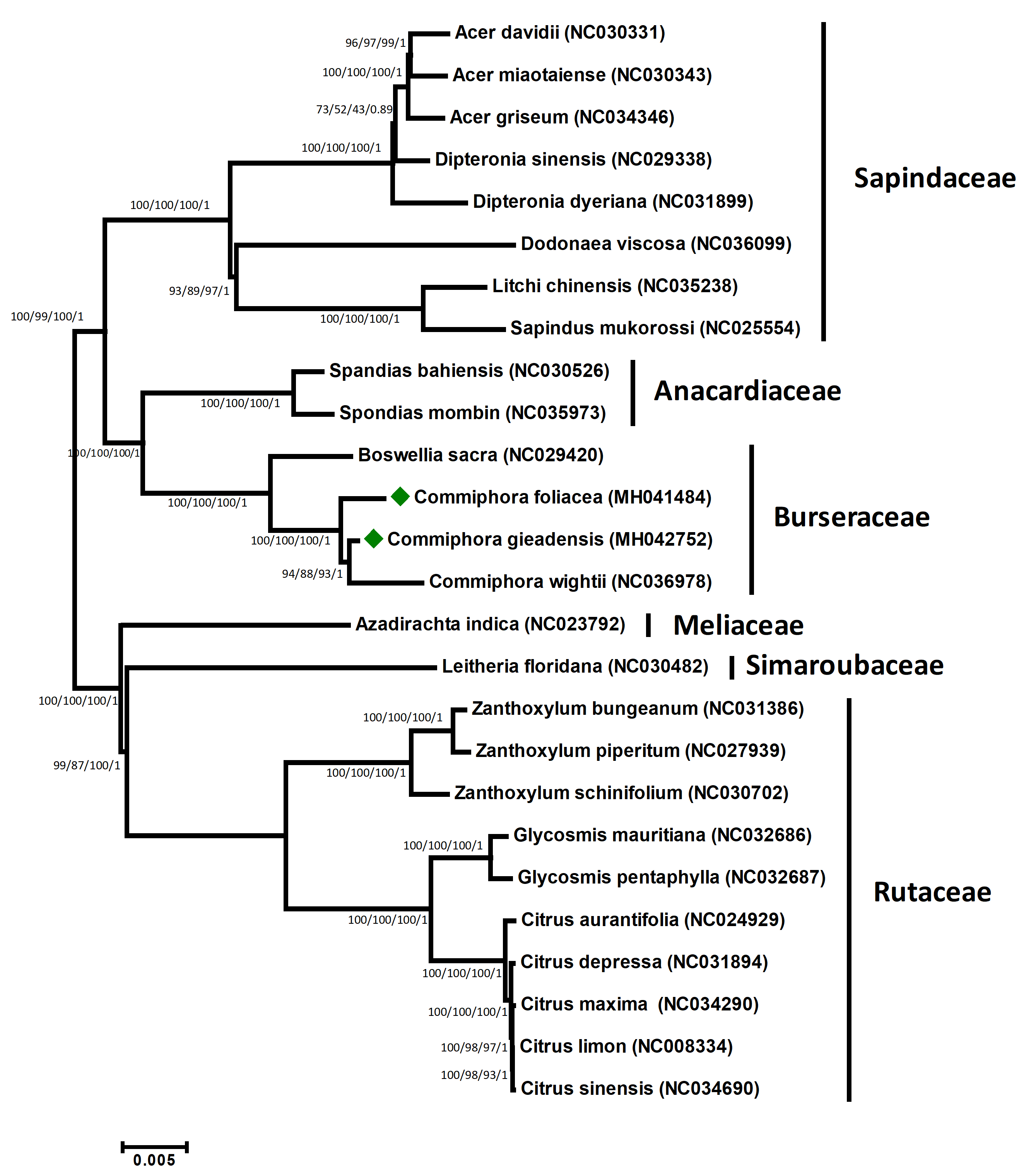

Supplement: S1 Fig — The 72 shared gene dataset was analyzed using four different methods: Bayesian inference (BI), maximum parsimony (MP), maximum likelihood (ML), and neighbor-joining (NJ). Numbers above the branches represent bootstrap values in the MP, ML, and NJ trees and posterior probabilities in the BI trees, whereas the number below the branches represents branch length. The red dot represents the position of C. gileadensis and C. foliacea. (TIF) [file pone.0208511.s001.tif]
