# Supplementary material for: First complete chloroplast genomics and comparative phylogenetic analysis of Commiphora gileadensis and C. foliacea: Myrrh producing trees
Source: PLoS One. 2019 Jan 10;14(1):e0208511. doi: 10.1371/journal.pone.0208511 (PMC6328178; doi:10.1371/journal.pone.0208511)
Supplement: S1 Table — (DOCX) [file pone.0208511.s002.docx]

**S1 Table. Genes in the sequenced *C. gileadensis* and *C. foliacea* chloroplast genome.**

| **Category** | **Group of genes** | **Name of genes** |
| --- | --- | --- |
| **Self-replication** | Large subunit of ribosomal proteins | *rpl2*, *14*, *16*, *20*, *22*, *23*, *32*, *33*, *36* |
|  | Small subunit of ribosomal proteins | *rps2, 3, 4, 7, 8, 11, 12, 14, ,16, 18, 19* |
|  | DNA dependent RNA polymerase | *rpoA, B, C1, C2* |
|  | rRNA genes | *rrn 4.5, rrn 5, rrn 16, rrn23* |
|  | tRNA genes | *trnA-UGC, trnC-GCA, trnD-GUC, trnE-UUC trnF-GAA, trnfM-CAU, trnG-UCC, trnH-GUG, trnI-CAU, trnI-GAU, trnK-UUU, trnL-CAA, trnL-UAA, trnL-UAG, trnM-CAU, trnN-GUU, trnP-GGG, trnP-UGG, trnQ-UUG, trnR-ACG, trnR-UCU, trnS-GCU, trnS-GGA, trnS-UGA, trnT-GGU, trnT-UGU, trnV-GAC, trnV-UAC, trnW-CCA, trnY-GUA* |
| **Photosynthesis** | Photosystem I | *psaA, B, C, I, J,* |
|  | Photosystem II | *psbA, B, C, D, E, F, H, I, J, K, L, M, N, T, Z* |
|  | Cytochrome b6/f complex | *petA, B, D, G, L, N* |
|  | ATP synthase | *atpA, B, E, F, H, I* |
|  | Rubisco | *rbcL* |
| **Other genes** |  |  |
|  | Maturase | *matK* |
|  | Protease | *clpP* |
|  | Envelop membrane protein | *cemA* |
|  | Subunit Acetyl- CoA-Carboxylate | *accD* |
|  | c-type cytochrome synthesis gene | *ccsA* |
| **Unknown** | Conserved Open reading frames | *ycf1,2, 3,4, 15,68* |
