# Supplementary material for: First complete chloroplast genomics and comparative phylogenetic analysis of Commiphora gileadensis and C. foliacea: Myrrh producing trees
Source: PLoS One. 2019 Jan 10;14(1):e0208511. doi: 10.1371/journal.pone.0208511 (PMC6328178; doi:10.1371/journal.pone.0208511)
Supplement: S2 Table — (DOCX) [file pone.0208511.s003.docx]

**S2 Table. Base compositions in *C. gileadensis* (C. g), *C. foliacea* (C. f), *C. wightii* (C. w) and *B. sacra* (B. s) cp genomes.**

|  | **T/U** | | | | **C** | | | | **A** | | | | **G** | | | | **Length (bp)** | | | |
| --- | --- | --- | --- | --- | --- | --- | --- | --- | --- | --- | --- | --- | --- | --- | --- | --- | --- | --- | --- | --- |
|  | *C*. *g* | *C*. *f* | CW | BS | *C*. *g* | *C*. *f* | CW | BS | *C*. *g* | *C*. *f* | CW | BS | *C*. *g* | *C*. *f* | CW | BS | *C*. *g* | *C*. *f* | CW | BS |
| **Genome** | 31.7 | 31.5 | 31.4 | 31.5 | 19.3 | 19.3 | 19.3 | 19.2 | 30.5 | 30.7 | 30.7 | 30.9 | 18.6 | 18.5 | 18.6 | 18.4 | 160268 | 160249 | 156064 | 160543 |
| **LSC** | 32.9 | 32.9 | - | 32.8 | 18.4 | 18.4 | - | 18.2 | 31.1 | 31.3 | -- | 31.6 | 17.5 | 18.4 | -- | 17.3 | 87885 | 87923 |  | 88054 |
| **SSC** | 34.3 | 33.4 | - | 33.9 | 16.9 | 16.8 | - | 16.8 | 33.4 | 34.4 | - | 34.1 | 15.4 | 15.6 | - | 15.3 | 18769 | 18746 |  | 18962 |
| **IR** | 28.6 | 28.5 | - | 28.6 | 22.1 | 22.1 | - | 20.8 | 28.4 | 28.6 | - | 28.5 | 20.8 | 20.8 | - | 22.1 | 26807 | 26790 |  | 26764 |
| **tRNA** | 25.5 | 25.4 | 25.3 | 25.3 | 23.0 | 23 | 23.1 | 23.2 | 22.4 | 22.3 | 22 | 22.2 | 29.1 | 29.2 | 29.6 | 29.3 | 2934 | 2934 | 2195 | 2919 |
| **rRNA** | 18.8 | 18.8 | 25.8 | 21.2 | 23.9 | 23.9 | 31 | 23.1 | 25.7 | 25.7 | 19.1 | 26.3 | 31.6 | 31.6 | 24. | 29.4 | 9050 | 9050 | 9394 | 9059 |
| **Protein Coding genes** | 30.8 | 31.3 | 31.5 | 31.1 | 18.1 | 18.2 | 18.0 | 18 | 30.7 | 29.8 | 29.4 | 30.5 | 20.4 | 20.7 | 21.1 | 20.5 | 78238 | 73119 | 60006 | 80361 |
| **1st position** | 24.806 | 24.330 | 23.172 | 23.582 | 18.720 | 18.787 | 18.953 | 19.039 | 29.860 | 30.812 | 29.222 | 30.560 | 24.296 | 25.491 | 28.652 | 26.880 | 26078 | 24373 | 20002 | 26770 |
| **2nd position** | 32.287 | 32.396 | 33.256 | 32.357 | 19.207 | 20.309 | 21.007 | 20.381 | 29.128 | 24.420 | 27.167 | 29.323 | 19.376 | 18.873 | 18.568 | 18.001 | 26078 | 24373 | 20002 | 26770 |
| **3rd position** | 35.294 | 37.102 | 37.941 | 37.280 | 16.404 | 15.480 | 14.118 | 14.643 | 30.707 | 30.304 | 31.636 | 31.606 | 17.593 | 17.113 | 16.003 | 16.553 | 26078 | 24373 | 20002 | 26770 |

*C. g= C. gileadensis, C. f = C. foliacea, C.w= C. wightii, B.s= B. sacra*
