# Supplementary material for: First complete chloroplast genomics and comparative phylogenetic analysis of Commiphora gileadensis and C. foliacea: Myrrh producing trees
Source: PLoS One. 2019 Jan 10;14(1):e0208511. doi: 10.1371/journal.pone.0208511 (PMC6328178; doi:10.1371/journal.pone.0208511)
Supplement: S3 Table — (DOCX) [file pone.0208511.s004.docx]

**S3 Table. The codon–anticodon recognition pattern and codon usage for the *C. gileadensis* chloroplast genome.**

| **Amino acid** | **Codon** | **No** | **RSCU** | **tRNA** | **Amino acid** | **Codon** | **No** | **RSCU** | **tRNA** |
| --- | --- | --- | --- | --- | --- | --- | --- | --- | --- |
| Phe | UUU | 2425 | 1.22 |  | Tyr | UAC | 708 | 0.66 | *trnY-GUA* |
| Phe | UUC | 1557 | 0.78 | *trnF-GAA* | Tyr | UAU | 1433 | 1.34 |  |
| Leu | UUA | 990 | 1.14 | *trnL-UAA* | Stop | UAA | 1133 | 1.22 |  |
| Leu | UUG | 1142 | 1.31 | *trnL-CAA* | Stop | UGA | 901 | 0.97 |  |
| Leu | CUU | 1206 | 1.38 |  | Stop | UAG | 751 | 0.81 |  |
| Leu | CUC | 695 | 0.80 |  | Cyc | UGC | 473 | 0.81 | *trnC-GCA* |
| Leu | CUA | 683 | 0.78 | *trnL-UAG* | Trp | UGG | 703 | 1.00 | *trnW-CCA* |
| Leu | CUG | 515 | 0.59 |  | His | CAU | 980 | 1.44 |  |
| Ile | AUU | 1799 | 1.22 |  | His | CAC | 383 | 0.56 | *trnH-GUG* |
| Ile | AUC | 1134 | 0.77 | *trnI-GAU* | Gln | CAA | 1093 | 1.37 | *trnQ-UUG* |
| Ile | AUA | 1495 | 1.01 | *trnI-CAU* | Gln | CAG | 497 | 0.63 |  |
| Met | AUG | 920 | 1.00 | *trn(f)M-CAU* | Asn | AAU | 1841 | 1.39 |  |
| Val | GUU | 807 | 1.37 |  | Asn | AAC | 799 | 0.61 | *trnN-GUU* |
| Val | GUC | 462 | 0.78 | *trnV-GAC* | Lys | AAA | 2141 | 1.34 | *trnK-UUU* |
| Val | GUA | 704 | 1.19 | *trnV-UAC* | Lys | AAG | 1058 | 0.66 |  |
| Val | GUG | 388 | 0.66 |  | Asp | GAU | 1213 | 1.47 |  |
| Ser | UCC | 911 | 1.13 | *trnS-GGA* | Asp | GAC | 439 | 0.53 | *trnD-GUC* |
| Ser | UCA | 885 | 1.10 | *trnS-UGA* | Glu | GAA | 1464 | 1.41 | *trnE-UUC* |
| Ser | UCG | 636 | 0.79 |  | Glu | GAG | 610 | 0.59 |  |
| Pro | CCU | 687 | 1.09 |  | Arg | CGU | 469 | 0.81 | *trnR-ACG* |
| Pro | CCC | 655 | 1.04 | *trnP-GGG* | Arg | CGC | 283 | 0.49 |  |
| Pro | CCA | 758 | 1.21 | *trnP-UGG* | Arg | CGA | 637 | 1.10 |  |
| Pro | CCG | 414 | 0.66 |  | Arg | CGG | 378 | 0.65 |  |
| Thr | ACU | 686 | 1.15 |  | Ser | AGU | 690 | 0.85 |  |
| Thr | ACC | 633 | 1.06 |  | Ser | AGC | 510 | 0.63 | *trnS-GCU* |
| Thr | ACA | 661 | 1.10 | *trnT-UGU* | Arg | AGA | 1047 | 1.81 | *trnR-UCU* |
| Thr | ACG | 415 | 0.69 |  | Arg | AGG | 654 | 1.13 |  |
| Ala | GCU | 531 | 1.33 |  | Gly | GGU | 605 | 1.02 |  |
| Ala | GCC | 366 | 0.92 |  | Gly | GGC | 398 | 0.67 | *trnG-GCC* |
| Ala | GCA | 445 | 1.11 | *trnA-UGC* | Gly | GGA | 786 | 1.33 | *trnG-UCC* |
| Ala | GCG | 256 | 0.64 |  | Gly | GGG | 578 | 0.98 |  |
