# Supplementary material for: First complete chloroplast genomics and comparative phylogenetic analysis of Commiphora gileadensis and C. foliacea: Myrrh producing trees
Source: PLoS One. 2019 Jan 10;14(1):e0208511. doi: 10.1371/journal.pone.0208511 (PMC6328178; doi:10.1371/journal.pone.0208511)
Supplement: S4 Table — (DOCX) [file pone.0208511.s005.docx]

**S4 Table. The codon–anticodon recognition pattern and codon usage for the *C. foliacea* chloroplast genome.**

| **Amino acid** | **Codon** | **No** | **RSCU** | **tRNA** | **Amino acid** | **Codon** | **No** | **RSCU** | **tRNA** |
| --- | --- | --- | --- | --- | --- | --- | --- | --- | --- |
| Phe | UUU | 2287 | 1.18 |  | Tyr | UAC | 708 | 0.65 | *trnY-GUA* |
| Phe | UUC | 1591 | 0.82 | *trnF-GAA* | Tyr | UAU | 1475 | 1.35 |  |
| Leu | UUA | 1099 | 1.28 | *trnL-UAA* | Stop | UAA | 1166 | 1.14 |  |
| Leu | UUG | 1108 | 1.29 | *trnL-CAA* | Stop | UGA | 1135 | 1.11 |  |
| Leu | CUU | 1082 | 1.26 |  | Stop | UAG | 759 | 0.74 |  |
| Leu | CUC | 640 | 0.75 |  | Cyc | UGC | 459 | 0.80 | *trnC-GCA* |
| Leu | CUA | 744 | 0.87 | *trnL-UAG* | Trp | UGG | 717 | 1.00 | *trnW-CCA* |
| Leu | CUG | 466 | 0.54 |  | His | CAU | 944 | 1.42 |  |
| Ile | AUU | 1787 | 1.21 |  | His | CAC | 383 | 0.58 | *trnH-GUG* |
| Ile | AUC | 1140 | 0.77 | *trnI-GAU* | Gln | CAA | 959 | 1.39 | *trnQ-UUG* |
| Ile | AUA | 1508 | 1.02 | *trnI-CAU* | Gln | CAG | 419 | 0.61 |  |
| Met | AUG | 864 | 1.00 | *trn(f)M-CAU* | Asn | AAU | 1798 | 1.38 |  |
| Val | GUU | 756 | 1.29 |  | Asn | AAC | 801 | 0.62 | *trnN-GUU* |
| Val | GUC | 462 | 0.79 | *trnV-GAC* | Lys | AAA | 2197 | 1.33 | *trnK-UUU* |
| Val | GUA | 708 | 1.21 | *trnV-UAC* | Lys | AAG | 1095 | 0.67 |  |
| Val | GUG | 418 | 0.71 |  | Asp | GAU | 1097 | 1.44 |  |
| Ser | UCC | 915 | 1.08 | *trnS-GGA* | Asp | GAC | 430 | 0.56 | *trnD-GUC* |
| Ser | UCA | 1025 | 1.21 | *trnS-UGA* | Glu | GAA | 1403 | 1.43 | *trnE-UUC* |
| Ser | UCG | 705 | 0.83 |  | Glu | GAG | 563 | 0.57 |  |
| Pro | CCU | 703 | 1.09 |  | Arg | CGU | 401 | 0.67 | *trnR-ACG* |
| Pro | CCC | 661 | 1.02 | *trnP-GGG* | Arg | CGC | 281 | 0.47 |  |
| Pro | CCA | 763 | 1.18 | *trnP-UGG* | Arg | CGA | 671 | 1.13 |  |
| Pro | CCG | 464 | 0.72 |  | Arg | CGG | 406 | 0.68 |  |
| Thr | ACU | 665 | 1.14 |  | Ser | AGU | 631 | 0.74 |  |
| Thr | ACC | 601 | 1.03 |  | Ser | AGC | 547 | 0.64 | *trnS-GCU* |
| Thr | ACA | 678 | 1.17 | *trnT-UGU* | Arg | AGA | 1160 | 1.95 | *trnR-UCU* |
| Thr | ACG | 383 | 0.66 |  | Arg | AGG | 651 | 1.09 |  |
| Ala | GCU | 548 | 1.33 |  | Gly | GGU | 562 | 0.97 |  |
| Ala | GCC | 398 | 0.96 |  | Gly | GGC | 366 | 0.63 | *trnG-GCC* |
| Ala | GCA | 474 | 1.15 | *trnA-UGC* | Gly | GGA | 818 | 1.41 | *trnG-UCC* |
| Ala | GCG | 233 | 0.56 |  | Gly | GGG | 581 | 1.00 |  |
