# Supplementary material for: First complete chloroplast genomics and comparative phylogenetic analysis of Commiphora gileadensis and C. foliacea: Myrrh producing trees
Source: PLoS One. 2019 Jan 10;14(1):e0208511. doi: 10.1371/journal.pone.0208511 (PMC6328178; doi:10.1371/journal.pone.0208511)
Supplement: S5 Table — (DOCX) [file pone.0208511.s006.docx]

**S5 Table. Simple sequence repeats (SSRs) in the *C. wightii* chloroplast genome.**

| **Unit** | **Length** | **No** | **SSR start** |
| --- | --- | --- | --- |
| **AT** | 14 | 1 | 9281 |
|  | 12 | 1 | 61697 |
|  | 11 | 3 | 32731, 118941,143477 |
|  | 10 | 2 | 20974,37018 |
|  | 9 | 10 | 32742, 33511, 37006, 52771,64086,65049,96233,112572,145290,154925 |
|  | 8 | 12 | 7807, 28561, 46643,57456, 61032, 61321,62007,66383,87095,114411,120991,155922 |
| **AG** | 10 | 1 | 63181 |
|  | 9 | 1 | 36801 |
|  | 8 | 12 | 49750,88210,88222,89199,91374,98527,108241,134002,150153,152328,153306,153318 |
| **A** | 20 | 2 | 84363, 126683, |
|  | 18 | 1 | 83290, |
|  | 17 | 1 | 4348 |
|  | 16 | 1 | 9029 |
|  | 14 | 2 | 4444,117428 |
|  | 13 | 2 | 61578,115688 |
|  | 12 | 6 | 9542, 14020,14076,73659,110382,131857 |
|  | 11 | 3 | 4336,69828,73647 |
|  | 10 | 13 | 1534,13915,13940,19494,33307,44828,50600,60866,62719,68282,80342,117443,127342 |
| **AAAG** | 13 |  | 121723 |
| **AAAT** | 16 | 1 | 68356 |
| **AGAT** | 12 | 1 | 36993 |
| **ACT** | 9 | 2 | 13483,59747 |
| **AAT** | 13 | 2 | 56399, 83826, |
|  | 12 | 2 | 50630, 127764 |
|  | 11 | 8 | 14760, 14979,53329,68584,72237,87380,114431,155635 |
|  | 10 | 4 | 69859, 112920,125588, |
|  | 9 |  | 4486,8977,10845,10874,16649,32225,38776,48602,48713,66086,77563,113853,129227 |
| **AAG** | 12 | 2 | 95526,145994, |
|  | 11 | 2 | 5433,76937, |
|  | 10 | 6 | 23237,89723,96790,122151,151802,155416, |
|  | 9 | 11 | 71522, 92057, 92163, 93902, 98231, 101976,127997 |
|  |  |  | 140226,147621,149360,149466 |
| **ATC** | 10 | 1 | 6535 |
|  | 9 | 1 | 154952 |
| **AAC** | 10 | 1 | 46447 |
|  | 9 | 5 | 15837,57123,76304,111775,130470, |
| **ACC** | 9 | 3 | 37883,92427,149096 |
| **AGC** | 9 | 6 | 42172,58534,86958,106683,123480,135559, |
| **AGG** | 9 | 2 | 104599, 137644 |
| **AAATT** | 15 | 1 | 23857 |
| **AAAGG** | 16 | 1 | 48008 |
| **AATG** | 13 | 1 | 127527 |
| **AATT** | 12 | 1 | 114456 |
