# Supplementary material for: First complete chloroplast genomics and comparative phylogenetic analysis of Commiphora gileadensis and C. foliacea: Myrrh producing trees
Source: PLoS One. 2019 Jan 10;14(1):e0208511. doi: 10.1371/journal.pone.0208511 (PMC6328178; doi:10.1371/journal.pone.0208511)
Supplement: S6 Table — (DOCX) [file pone.0208511.s007.docx]

**S6 Table. Simple sequence repeats (SSRs) in *C. gileadensis* chloroplast genome.**

| **Unit** | **Length** | **No** | **SSR start** |
| --- | --- | --- | --- |
| **AT** | 14 | 1 | 9916 |
|  | 12 | 1 | 62945 |
|  | 11 | 3 | 9931,33960,121990 |
|  | 10 | 3 | 9949,21753,38275 |
|  | 9 | 9 | 33971,34777,54058,65727,89022,98702,115230,149445,159125 |
|  | 8 | 11 | 8426, 29366, 48011, 58761, 62572,63255, 67096, 88023,117074, 124048, 160125 |
| **AG** | 10 | 1 | 64822 |
|  | 9 | 1 | 38070 |
|  | 8 | 13 | 51004,90630,90642,91622,93799,99471,110731,137417,148677,154349,156526,157506,157518 |
| **A** | 18 | 1 | 84221, |
|  | 17 | 1 | 130416 |
|  | 16 | 4 | 30981, 64342, 118437, 118744 |
|  | 15 | 1 | 53314 |
|  | 14 | 1 | 51872 |
|  | 13 | 3 | 72352, 117107, 120577 |
|  | 12 | 10 | 1776, 9664, 29608, 38912, 46158, 50881, 63850, 74519, 74547, 117750 |
|  | 11 | 9 | 6023, 14794, 31266,32803, 51684, 58321, 74503, 75558,88382 |
|  | 10 | 45 | 2419, 3796, 4755, 4948, 6079,8782, 13260, 13855,14644,15102,20272,24911,31925,34128,34543 ,38837,45592,47448,54746, 57645, 58212,67220, 69203,70356,70670,70899,74288,74715,75529,81235,87652, 112876, 117181, 117201,120463,122216,123523, 131257, 132865, 135270, |
| **C** | 10 | 5 | 36973, 102363, 104917, 143229, 145783 |
| **AAAG** | 13 | 1 | 124778 |
| **AGAT** | 12 | 1 | 38262 |
| **AAGT** | 12 | 1 | 80828 |
| **ACT** | 9 | 2 | 14211, 61051 |
| **AAT** | 13 | 2 | 57699, 84747, |
|  | 12 | 2 | 51910, 130846, |
|  | 11 | 7 | 15504, 15723, 69421, 73089,88308, 117095,159837 |
|  | 10 | 5 | 7260, 54631,70700,115580,128647 |
|  | 9 | 9 | 9612,11585,17420,33454,40036,66781,78454,116515,132316, |
| **AAG** | 12 | 2 | 97995,150149, |
|  | 11 | 1 | 77811 |
|  | 10 | 8 | 24018,88530,92146,101206,125206,146940,156000,159616 |
|  | 9 | 12 | 72373,94482,94588,96360,99766,104457,131080,143690,148381,151787,153559,153665 |
| **ATC** | 10 | 1 | 7144, |
|  | 9 | 2 | 88995,159152 |
| **AAC** | 10 | 1 | 47814 |
| **ACC** | 9 | 3 | 39122,94852, 153295 |
| **AGC** | 9 | 6 | 43436,59839, 87886,109173,126536,138974, |
| **AAAC** | 16 | 1 | 132525 |
| **AATT** | 15 | 1 | 39405 |
|  | 12 | 1 | 117123 |
| **AAAGG** | 16 | 1 | 49378 |
| **AAAGG** | 16 | 1 | 49378 |
| **AAATT** | 15 | 1 | 24646 |
| **AAAAT** | 15 | 1 | 691177 |
