# Supplementary material for: First complete chloroplast genomics and comparative phylogenetic analysis of Commiphora gileadensis and C. foliacea: Myrrh producing trees
Source: PLoS One. 2019 Jan 10;14(1):e0208511. doi: 10.1371/journal.pone.0208511 (PMC6328178; doi:10.1371/journal.pone.0208511)
Supplement: S7 Table — (DOCX) [file pone.0208511.s008.docx]

**S7 Table. Simple sequence repeats (SSRs) in *C. foliacea* chloroplast genome.**

| **Unit** | **Length** | **No** | **SSR start** |
| --- | --- | --- | --- |
| **AC** | 8 | 1 | 28279 |
| **AT** | 20 | 1 | 9863 |
|  | 14 | 1 | 63088 |
|  | 11 | 2 | 33905,122019, |
|  | 10 | 2 | 9888,21769, |
| **AG** | 10 | 1 | 64957, |
|  | 9 | 1 | 38015 |
|  | 8 | 12 | 90667, 90679,91657,93833,99504,110752,137414,148662,154333,156509,157487,157499, |
| **A** | 18 | 1 | 14759 |
|  | 14 | 2 | 1774,51985, |
|  | 13 | 4 | 47393,64481,70466,74616 |
|  | 12 | 3 | 71250, 74820,122243 |
|  | 11 | 15 | 9556, 9706, 13196, 20287, 38782, 49998, 50991, 58434, 69288, 69414, 72463, 112894, 130431, 133391, 135269, |
|  | 10 | 23 | 4753,6837,7125,8674,13797,29626,34486,38407,45543,46108,53426, 57758,58325, 62972, 63996,71009,74402,74931, 79519, 84263,117806,120479,123551 |
| **C** | 10 | 3 | 36918,102395,145769 |
| **AAAG** | 13 | 1 | 124800 |
| **AGAT** | 12 | 1 | 38207 |
| **AAGT** | 12 | 1 | 80871 |
| **AAT** | 13 | 3 | 57812, 84791,52023 |
|  | 12 | 2 | 117148, 130856 |
|  | 11 | 7 | 15473,15692,54745,69530,73203,88346,159817 |
|  | 10 | 4 | 7168,70810,115636,128664 |
|  | 9 | 11 | 9504,11515,17437,33399,39982,49929,50046,66916,78547,116569,132321, |
| **AAG** | 12 | 2 | 98029,150133, |
|  | 11 | 1 | 77903 |
|  | 10 | 8 | 24033,88567,92181,101239,125228,146925,155983,159597 |
|  | 9 | 12 | 72482, 94516, 94622,96394,99799,104480,131090,143685,148366,151771, 153543,153649 |
| **ATC** | 10 | 1 | 7051 |
|  | 9 | 2 | 89032,159133 |
| **AAC** | 10 | 1 | 47762, |
|  | 9 | 5 | 16624,58541,77270,114266,133899, |
| **AGC** | 9 | 5 | 43379,59952,109194,126553,138971, |
| **ACC** | 9 | 3 | 39067,94886,153279 |
| **AGG** | 9 | 2 | 107106,141059 |
| **AAAGG** | 16 | 1 | 49332 |
| **AAATT** | 15 | 1 | 24660 |
| **AATG** | 13 | 1 | 130619 |
| **AATT** | 15 | 1 | 39351 |
|  | 12 | 1 | 117174 |
