# Supplementary material for: First complete chloroplast genomics and comparative phylogenetic analysis of Commiphora gileadensis and C. foliacea: Myrrh producing trees
Source: PLoS One. 2019 Jan 10;14(1):e0208511. doi: 10.1371/journal.pone.0208511 (PMC6328178; doi:10.1371/journal.pone.0208511)
Supplement: S8 Table — (DOCX) [file pone.0208511.s009.docx]

**S8 Table. Simple sequence repeats (SSRs) in *Boswellia sacra* chloroplast genome*.***

| **Unit** | **Length** | **No** | **SSR start** |
| --- | --- | --- | --- |
| **A** | 20 | 1 | 58455 |
|  | 19 | 1 | 130158 |
|  | 18 | 2 | 14734, 84385 |
|  | 17 | 2 | 4679, 9516 |
|  | 16 | 1 | 63188 |
|  | 15 | 1 | 118676 |
|  | 14 | 1 | 120795 |
|  | 12 | 7 | 10196, 14690, 29019, 45696, 74909, 112968, 135620 |
|  | 11 | 12 | 4566, 4578, 9673, 38927, 58428, 64287, 64661, 67202, 71347, 74711, 117641, 119182 |
|  | 10 | 28 | 1769, 5860, 6973, 7939, 10686, 14590, 14615, 20253, 29239, 29906, 31442, 34648, 46209, 49936, 57871, 71109, 71647, 74497, 79313, 81434, 113032, 114850, 117470, 118446, 120810, 122533, 130823, 135558 |
| **AT** | 12 | 3 | 49380, 72021, 118991 |
|  | 11 | 3 | 50086, 120682, 122312 |
|  | 10 | 3 | 21733, 50519, 63260, |
|  | 9 | 8 | 9899 ,34079, 34857, 66023, 89191, 98820,149771, 159400 |
|  | 8 | 16 | 8312, 29330, 45885, 48039, 51468, 55534, 58977,62558, 62885, 63562, 67356, 70572, 79227, 88193, 124361, 160339. |
| **AG** | 10 | 1 | 65118 |
|  | 9 | 3 | 38146, 128919, 133069 |
|  | 8 | 14 | 59161, 90779, 90811, 91798,93968, 99588, 110826, 132830, 137766,149004, 154624, 156794, 157781, 157793 |
| **AAT** | 14 | 1 | 54843 |
|  | 13 | 2 | 57925, 70908 |
|  | 12 | 3 | 50027, 52097,131240 |
|  | 11 | 7 | 9776, 15427, 15649, 33553,88478,117143, 160111, |
|  | 10 | 2 | 30694, 128959, |
|  | 9 | 15 | 394, 9465, 11505, 11536, 14773, 15968, 17409,38947, 40124, 69627, 114962, 115632,116565, 118000,132959, |
| **AAG** | 12 | 2 | 98114, 150474 |
|  | 11 | 2 | 50436, 78020 |
|  | 10 | 9 | 23996, 52782, 88699, 92322, 101323,125518,147267, 156268, 159891 |
|  | 9 | 9 | 94645,94751, 96490, 99883, 131474, 148708,152101,153840, 153946. |
| **ATC** | 9 | 2 | 89164, 159427 |
| **AAC** | 10 | 1 | 47842 |
|  | 9 | 5 | 16595, 58644, 77387,114366, 134225, |
| **ACT** | 9 | 2 | 61265, 120081 |
| **AGC** | 9 | 6 | 43520, 60055, 88056, 109268, 126847, 139323 |
| **ACC** | 9 | 3 | 39235, 95015, 153576 |
| **AAAG** | 14 | 2 | 51864, 125090 |
| **AGAT** | 14 | 1 | 34089 |
|  | 12 | 1 | 38353 |
| **AAAT** | 13 | 1 | 6383 |
| **AATT** | 12 | 1 | 117168 |
| **AATG** | 13 | 1 | 131003 |
| **AAG** | 14 | 1 | 51864 |
|  | 13 | 1 | 125090 |
| **AATATG** | 20 | 1 | 118904 |
| **AATATG** | 20 | 1 | 118904 |
